# Rare paratesticular localization of dedifferentiated liposarcoma: Case report and review of the literature

**DOI:** 10.1097/MD.0000000000033265

**Published:** 2023-03-17

**Authors:** Mariana Deacu, Mariana Aschie, Madalina Bosoteanu, Sorin Vamesu, Gabriela-Izabela Baltatescu, Georgeta Camelia Cozaru, Cristian Ionut Orasanu, Raluca Ioana Voda

**Affiliations:** a Clinical Service of Pathology, Departments of Pathology, “Sf. Apostol Andrei” Emergency County Hospital, Constanţa, Romania; b Department of Pathology, Faculty of Medicine, “Ovidius” University of Constanţa, Constanţa, Romania; c Academy of Medical Sciences of Romania, Bucharest, Romania; d Center for Research and Development of the Morphological and Genetic Studies of Malignant Pathology-CEDMOG, “Ovidius” University of Constanţa, Constanţa, Romania; e Clinical Service of Pathology, Departments of Genetics, “Sf. Apostol Andrei” Emergency County Hospital, Constanta, Romania.

**Keywords:** dedifferentiation, liposarcoma, MDM2, metastasis, paratesticular, spermatic cord

## Abstract

Dedifferentiated liposarcoma is defined as a malignant tumor that changes its shape from a well-differentiated liposarcoma to a non-liposarcomatous form. Most paratesticular liposarcomas manifest as an inguinal, painless, slow-growing mass. The standard treatment is extensive surgical excision, radiotherapy being proposed for cases with positive margins, those with recurrence, or in cases of the existence of unfavorable prognostic factors.

**Patient concerns:**

We present the case of a young patient diagnosed initially with left hydrocele, which after 2 years proved to mask a differentiated liposarcoma of the spermatic cord. The initial clinical manifestations were represented by the increase in volume of the left groin-scrotal region and pain at this level.

**Diagnosis:**

Microscopic examination in hematoxylin-eosin staining highlighted the presence of lipoblasts and fibroblasts in association with areas of hemorrhage and tumor necrosis. The performed immunohistochemical tests confirmed the diagnosis of dedifferentiated liposarcoma. To support and confirm the presence of the mouse double minute 2 homolog gene mutation, chromogenic in situ hybridization analysis was performed.

**Interventions:**

The initial treatment was the surgical one. After 2 weeks, the patient received zolendronic acid for hypercalcemia which was caused by the osseous metastasis.

**Outcomes:**

The patient died secondary to acute renal failure caused by hypercalcemia despite the treatment received.

**Lessons:**

This case underlines the importance of both the correct management of oncological patients, as well as immunohistochemical and genetic tests in the identification of prognostic factors, with the ultimate goal of administering an appropriate oncological treatment.

## 1. Introduction

In adults, over 75% of primary paratesticular tumors form in the spermatic cord and represent 7 to 10% of all intrascrotal tumors. Of these, 20% are liposarcomas.^[1]^ Liposarcoma is a tumor originating in the mesenchymal cells, the most frequent location being the retroperitoneal one. Other rare locations are spermatic cord, mediastinum, head and neck, and trunk. According to the latest edition of the World Health Organization (2020), liposarcoma is classified as well-differentiated, dedifferentiated, myxoid, pleomorphic, and myxoid pleomorphic liposarcoma.^[2]^ Most paratesticular liposarcomas manifest as a painless, slow-growing inguinal mass. These tumors are often misdiagnosed as hydrocele, lipoma, funicular cyst, testicular tumor, or inguinoscrotal hernia.^[3]^ The standard treatment is extensive surgical excision, radiotherapy being proposed for cases with positive margins, those with recurrence or if they are associated with negative prognostic factors.^[4]^

## 2. Case report

### 2.1. Clinical summary

A 42-year-old patient presented to the hospital for an increase in the volume of the left inguinal-scrotal region and pain at this level. Two years before presenting to the hospital, he was diagnosed with a minimal amount of left hydrocele, which is why he did not undergo any treatment for this pathology. On palpation, a painful fluid collection was identified. Paraclinically, carcinoembryonic antigen and human chorionic gonadotropin had normal values. Imagistically, computed tomography highlighted a left scrotal cavity with an enlarged projection area with heterogeneous content by the presence of a fluid accumulation and a relatively well-defined nodular lesion with polylobate contour, native hypodense, with heterogeneous structure by the presence of non-capturing hypodense areas and isodense, iodophilic areas with vascular pathways included. Numerous areas of secondary determinations of osteolysis have also been identified, as well as paraaortic lymphadenopathy with adenopathic block formation.

**Figure 1.**
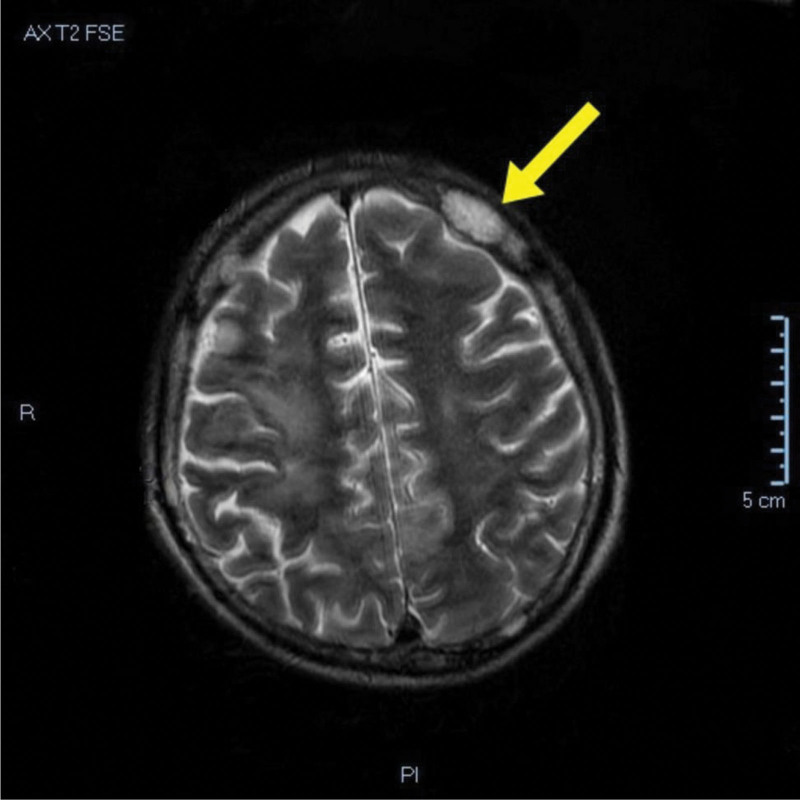
MRI aspect of bone metastasis: nodular lesion with hyperintensity in T2-weighted image. MRI = magnetic resonance imaging.

The patient was treated surgically by the resection of the left inguinoscrotal region without excision of the adenopathies and discharged in a good clinical state. After 2 weeks, the patient returned to the hospital with tonic-clonic seizures caused by hypercalcemia (serum calcium 17.6 mg/dL; ionized calcium 9 mg/dL). Due to the manifestations at the time of hospitalization, magnetic resonance imaging (MRI) was performed. It highlighted the presence of metastases at the level of the frontal bone. The MRI examination identified a relatively well-defined nodular lesion, with a maximum diameter of 2 cm and hyperintensity in T2-weighted image (Fig. 1). The patient received treatment with zolendronic acid (4 mg, intravenously, unique dose) to decrease bone demineralization, but he died during hospitalization due to acute kidney failure after 1 week from the seizure. The tumorectomy was sent for examination to the hospital’s pathology department.

### 2.2. Pathology findings

**Figure 2.**
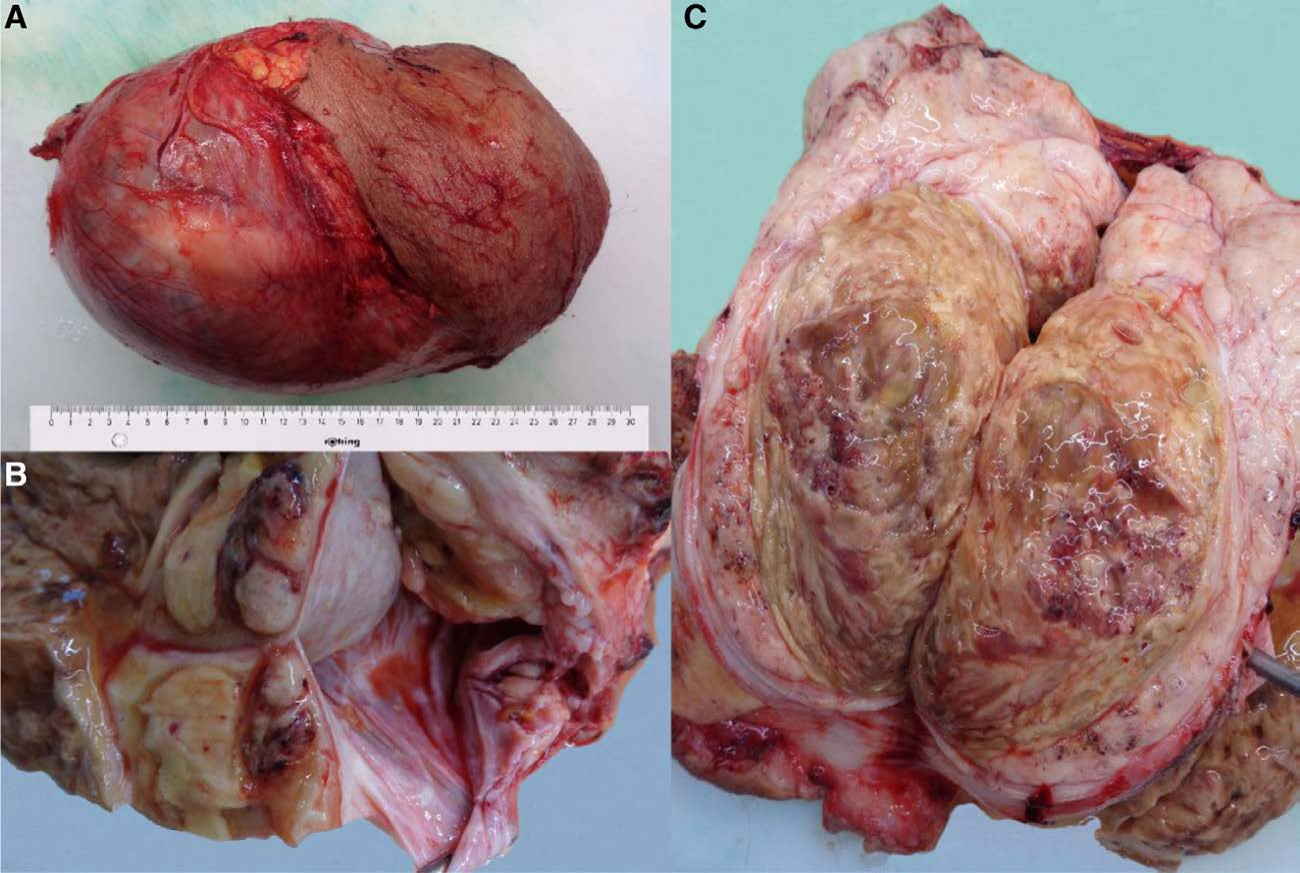
Macroscopic aspects of the tumor: (A) Nodular lesion partially covered with an edematous, adherent skin flap. (B) Cavity between the vagina and the albuginea, with a maximum diameter of 7 cm filled with a sero-citrin liquid. (C) Compact mass with white-gray appearance and areas of hemorrhage and necrosis.

Macroscopically, a nodular lesion with a mass of 3430 g and a maximum diameter of 29 cm was identified. It was partially covered with an edematous, adherent skin flap, which did not present retracted areas or scars (Fig. 2A). On cut-section was observed a cavity between the tunica vaginalis and albuginea, with a maximum diameter of 7 cm and a sero-citrine liquid content (Fig. 2B). The nodular lesion had a maximum diameter of 18 cm and a compact, white-gray appearance, with yellow-orange areas and cystic spaces. Areas of hemorrhage and necrosis have been identified inside the tumor mass (Fig. 2C). At the periphery of the lesion, testicular parenchyma with a maximum diameter of 4 cm was identified. It had a brown color and increased consistency being covered by a thickened albuginea tunic.

**Figure 3.**
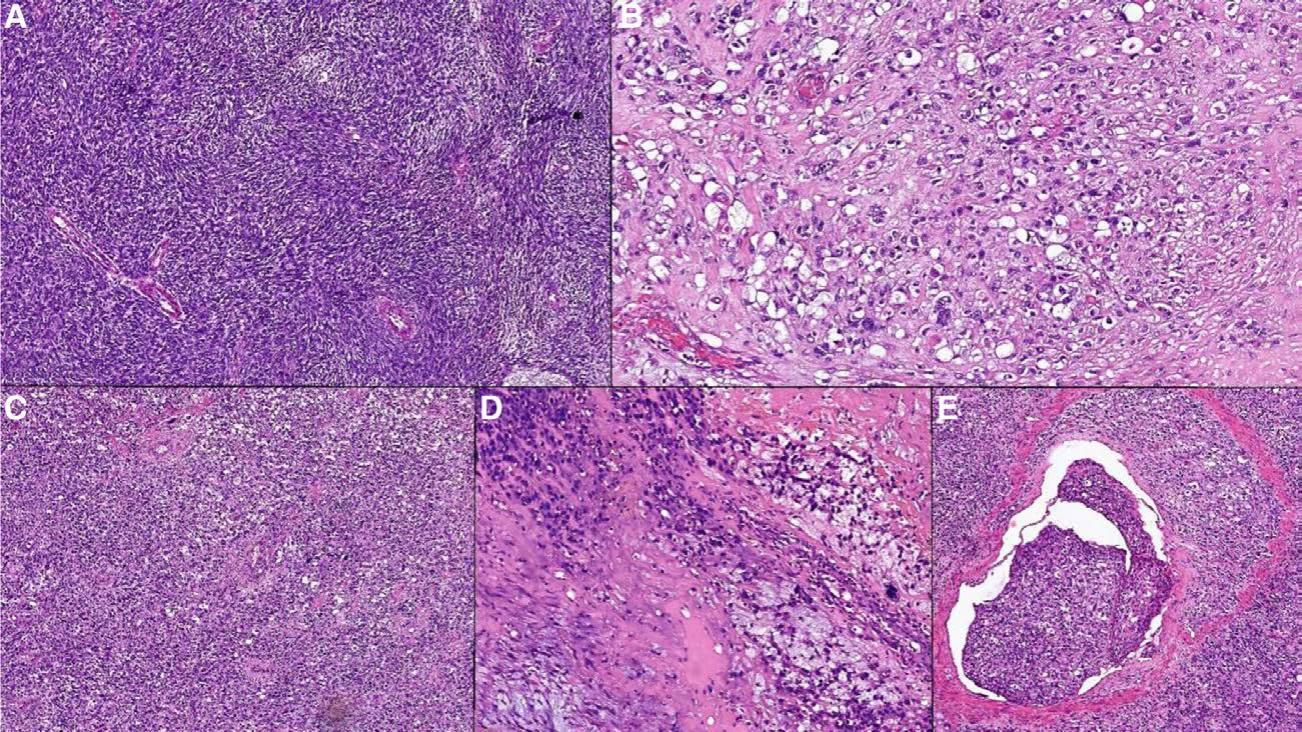
Examination on hematoxilin-eozin stain: (A) “Herringbone” pattern (Ob. 100x). (B) Area with adipose appearance with the presence of lipoblasts (Ob. 100x). (C) Solid area (Ob. 100x). (D) Solid area with atypical lipoblastic cells (Ob. 100x). (E) Tumoral embolus (Ob. 200x).

Microscopic examination on the hematoxylin-eosin stain revealed areas of fibroblast cells with reduced pleomorphism, “herringbone” pattern, and thickened wall vessels. Areas with varied aspects were identified. Among them are identified areas with adipose appearance and the presence of lipoblasts. Also, areas with myomatous differentiation with moderate atypia and areas with solid disposition, with marked nuclear pleomorphism and giant multinucleated tumor cells were observed (Fig. 3A–D). Vascular emboli are associated, including in the arterial vessels (Fig. 3E). The remaining testicular parenchyma shows fibrohyalinization and atrophy of the seminiferous tubules. Tumor proliferation did not invade the testicle and the overlying skin.

The Fédération Nationale des Centres de Lutte Contre Le Cancer grading was done as follows: tumor differentiation – 3 points, mitotic number – 2 points, tumor necrosis – 3 points. After adding the 3 values, we obtained a final score of 8 points, which corresponds to grade 3. Taking into account the presence of distant metastases at the time of diagnosis and the maximum diameter of the tumor (18 cm), the pTNM staging was: pT4 pM1. The patient was in stage IV of the disease. Considering the fact that the lymph nodes were not sent for examination, their invasions could not be assessed and, as a result, reported.

**Figure 4.**
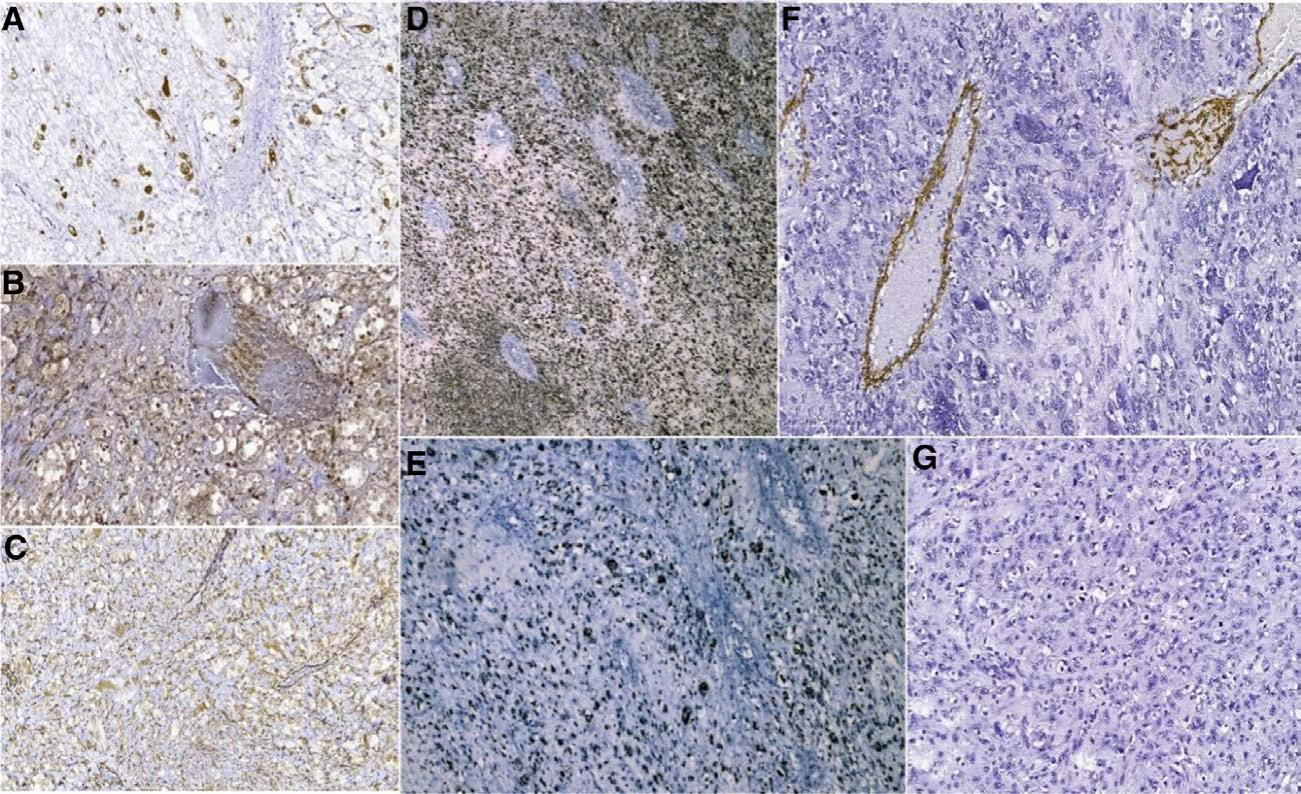
Immunohistochemical results: (A) Intense, diffuse positive nuclear reaction of Ki-67 (Ob. 200x). (B) Intense, diffuse positive nuclear reaction of MDM2 in lipoblastic cells (Ob. 200x). (C) Intense, diffuse positive cytoplasmatic reaction of Vimentin (Ob. 200x). (D) Intense, diffuse positive nuclear reaction of p16 (Ob. 200x). (E) Intense, diffuse positive nuclear reaction of p53 (Ob 200x). (F) Negative reaction of SMA (Ob. 200x). (G) Negative, reaction of GFAP (Ob. 200x). MDM2 = mouse double minute 2 homolog. GFAP = glial fibrillary acidic protein, SMA = smooth muscle actin.

The following immunohistochemical markers were performed: Ki-67, mouse double minute 2 homolog (MDM2), glial fibrillary acidic protein, smooth muscle actin, vimentin, p16, and p53 (Fig. 4). The immunohistochemical tests had the following results: intense and diffuse positive nuclear reaction in 60% of the nuclei for Ki-67, intense and diffuse positive nuclear reaction in lipoblastic tumor cells for MDM2, intense and diffuse positive nuclear reaction in 90% of the nuclei (overexpression pattern) for p53, intense and diffuse positive nuclear reaction in tumor cells for p16, intense and diffuse cytoplasmic positive reaction in tumor cells for vimentin, and negative reaction for glial fibrillary acidic protein and smooth muscle actin.

**Figure 5.**
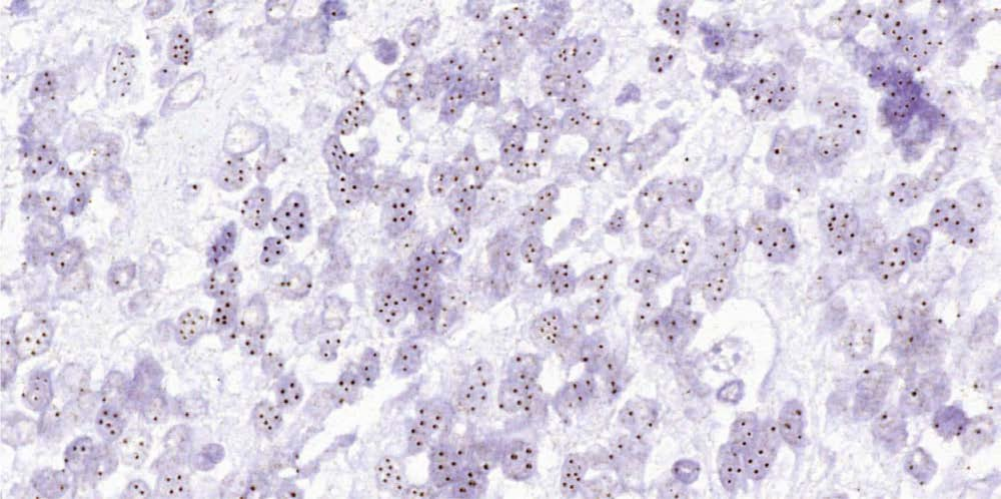
Representative photomicrograph of CISH analysis of MDM2 gene (brown signals) in a paraffine section, showing high degree amplification of MDM2 gene copies. CISH = chromogenic in situ hybridization, MDM2 = mouse double minute 2 homolog.

Genetic testing by chromogenic in situ hybridization (CISH) of the examined cells (x400 magnification) showed nuclear 4 to 8 individually arranged chromogenic signals, with an average of 5.48 on 50 cells. The examined cells revealed the presence of a high degree amplification of the MDM2 gene (Fig. 5).

The final diagnosis could only be established after the immunohistochemical examination, which was dedifferentiated liposarcoma. The diagnosis was confirmed with the help of a genetic examination by CISH.

## 3. Discussions

Soft tissue sarcomas are a rare disease, accounting for approximately 1% of all adult malignancies and approximately 20% of mesenchymal malignancies.^[5,6]^ Paratesticular sarcomas are defined as tumors originating in the scrotum and include the epididymis (4%), spermatic cord (76%), and vaginal tunic (20%). It is difficult to identify the origin given the size of the tumor and the degree of adhesion.^[7]^ Most occur in adults between the ages of 50 and 60.^[5]^ Data on the exact pathogenic mechanism are scarce, but it is known to be formed de novo from connective tissue and not by malignant transformation of a preexisting lipoma.^[8]^

The typical clinical presentation is inguinal edema or unilateral scrotal mass, which may or may not be painful, occasionally accompanied by hydrocele. Due to nonspecific manifestations, it is mandatory to differentiate preoperatively by inguinal hernia, hematocele, hydrocele, lipoma, epididymitis or orchiepididymitis, tuberculosis, and malignant lesions of the testicle.^[9]^ In general, these tumors are asymptomatic for many years, with symptoms lasting between 1 week and 5 years.^[5]^

In our case, it is a 42-year-old man, whose manifestations began at the age of 40, a decade earlier than the average age cited in the literature. His symptoms were initially represented by hydrocele, evolving in the next 2 years until the significant increase in the volume of the left inguinoscrotal region, and pain. Distant metastases were also present at the time of diagnosis.

Usually, the inguinal and scrotal masses are evaluated by ultrasound to differentiate the intratesticular location from the extratesticular one and the solid masses from the cystic ones.^[5]^ The typical appearance on ultrasound is represented by hypervascular and heterogeneous with hyperechoic areas, depending on the amounts of intratumoral adipose tissue, but it is not possible to differentiate between a lipoma and a liposarcoma, especially if it is well-differentiated.^[3,8]^ Computer tomography with contrast substance is the most used and most useful primary imaging method to diagnose a possible soft tissue sarcoma developed in the trunk and pelvis. Magnetic resonance imaging is reserved for patients allergic to iodinated contrast agents.^[2]^ Also, CT is useful for establishing localization, staging, and post-therapeutic follow-up.^[8]^

In our case, because the suspected diagnosis was hydrocele, a CT was performed. It revealed a nodular lesion with a heterogeneous structure by the presence of non-capturing hypodense areas and isodense, iodophilic areas. The presumptive diagnosis was liposarcoma. The imaging appearance of the inguinal-scrotal region in association with adenopathies and secondary bone findings led to the establishment of the initial surgical treatment: the resection of the left inguinoscrotal region without the excision of the lymph nodes.

Radical orchiectomy with high ligation of the spermatic cord, without positive microscopic edges, is the essential surgical treatment.^[2,10]^ Retroperitoneal lymph node dissection is usually performed in cases with present metastases.^[8]^ In Kamitani’s study, he showed that the 3-year survival rate without recurrence was significantly higher in patients treated with high inguinal orchiectomy (79.8%) than in those treated with tumorectomy alone (54.1%). The 3-year survival rate was 54.2% for those with positive margins and 88.6% for the cases with negative margins.^[11]^

Due to the increased risk of recurrence after surgical treatment, there is evidence that all spermatic cord tumors should be treated with adjuvant radiotherapy, regardless of histological grade and type.^[3]^

Some studies have shown that postoperative radiation therapy can significantly reduce the recurrence rate at 10 years in patients with high-grade tumors. It is recommended in cases of high-grade tumors, lymphatic invasion, positive margins, or relapses. Regarding chemotherapy, the data in the literature are limited, without a definitive role in the management of liposarcomas.^[5]^ Chemotherapeutic treatment is initiated with anthracyclines, usually doxorubicin in association with ifosfamide. However, dedifferentiated liposarcoma shows chemoresistance, with radiographic response in less than a third of patients.^[12]^

MDM2 is a major target in oncology treatment, with substances blocking the interaction between MDM2 and p53, restoring the wild-type activity of p53. One of these molecules is Nutlin-3A, which inhibits the interaction between the 2 genes and stabilizes p53, the disadvantage being the very low clinical efficacy and the limitation of the therapeutic dose secondary to severe thrombocytopenia.^[13]^

Dedifferentiated liposarcoma is a malignant tumor that changes its shape from a well-differentiated liposarcoma to a non-liposarcomatous form. Dedifferentiation is a time-dependent process in 10 to 15% of well-differentiated forms, the average period being 7.7 years, and the 5-year survival rate is 28%.^[7]^ The dedifferentiated subtype is the most worrying variant in terms of aggressive evolution and early recurrence.^[14]^ The areas of dedifferentiation have various aspects, but most frequently resemble undifferentiated pleomorphic sarcoma and high-grade or intermediate-grade myxofibrosarcoma.^[2]^ In our case, areas with a fascicular appearance, with a “Herringbone” pattern, accentuated pleomorphism and the presence of multinucleated giant cells were identified.

From a cytogenetic point of view, dedifferentiated liposarcoma is characterized by a supernumerary ring and giant marker chromosomes. They contain amplified seventy 12q13-15, but also other co-amplified chromosomal regions. Region 12q13-15 includes genes such as MDM2, CDK4, and HMGA2. MDM2 binds to p53 and regulates it negatively by preventing nuclear transcription and translocation, but also by supporting its degradation through ubiquitin E3 binding. Currently, immunohistochemical evaluation of MDM2 and CDK4 may help screen for 12q13-15 amplification.^[15]^ In our case, both the immunohistochemical expression and the amplification of the MDM2 gene confirmed the diagnosis of dedifferentiated liposarcoma.

The epigenomic initiators of dedifferentiation are poorly known, and more studies are needed. The molecular pathogenesis of dedifferentiation is a poorly understood process, although the genomic profile of this tumor is characterized by a low mutational burden. The amount of 12q13-15 amplification and MDM2 upregulation seems to be associated with the degree of dedifferentiation.^[12]^ Horvai et al observed that dedifferentiated liposarcomas have a higher total number of amplification.^[16]^

The tumor suppressor protein p53 is an essential regulator of cell division, DNA repair, apoptosis, and cellular senescence. The activity of this protein is primarily regulated by MDM2. Thus, the amplification of MDM2 leads to a decrease in p53 activity.^[12]^ P53 mutations were associated with the dedifferentiation process from well-differentiated to dedifferentiated liposarcoma. The location of the tumor has an important role in this process. In retroperitoneal localization, MDM2 amplifications and p53 mutations are mutually exclusive, but in non-retroperitoneal localizations of dedifferentiated liposarcoma, TP53 mutations appear in the presence of MDM2 amplification.^[17]^ The MDM2-p53 axis is regulated by multiple oncogenic signaling pathways involved in sarcomogenesis and represents a possible therapeutic target. Also, inhibitors of the co-activators of the MDM2-p53 pathway, such as inhibitors of mTOR, were studied. Most of the targeted therapies studied for dedifferentiated liposarcoma had clinically inferior results compared to anthracycline-based chemotherapy. For this reason, most of the studied inhibitors are not recommended as adjuvants to systemic therapy.^[12]^

Rekhi B et al studied the expression of p16 in 16 cases of dedifferentiated liposarcoma, all having a diffusely positive immunoexpression.^[18]^ According to Cai D et al, genetic mutations of p16 led to a decrease in overall survival. The high frequency of p53 gene mutations tends to determine the decrease in disease-free survival in sarcomas.^[19]^

In our case, we identified a large number of amplifications of the MDM2 gene. We also observed the presence of p53 gene mutation and p16 overexpression. This association justifies the fulminant evolution of the presented case, the death occurring within 3 weeks from the time of diagnosis.

The main way of metastasis of such a tumor is by contiguous extension through the inguinal canal into the abdominal cavity, the extension by hematogenous and lymphatic route being rarer.^[10]^ In the study by Ballo et al 5-year recurrence rates at the loco-regional, hematogenous, and pelvic lymph nodes were 19.4%, 11.1%, and respectively 5.5%.^[20]^

In the case of our patient, he presented at the time of diagnosis bone metastases that occur by hematogenous route, but also secondary lymph nodes involvement. Contiguous extension at the level of the adjacent organs was not identified.

Negative margins are an essential factor for non-recurrent survival.^[14]^ Wang et al observed that age <50 and incomplete surgical resection are predictive factors of non-recurrent survival and overall survival.^[9]^ Contrary, Dotan et al reported tumor size and the absence of metastases at the time of diagnosis are the only significant predictors of disease-specific survival.^[21]^ Stojadinovic et al also claim that positive surgical margins significantly increase the risk of local recurrence in addition to increasing the risk of metastasis and disease-related death.^[22]^

The postoperative and post-diagnostic management consists of imaging follow-up performed at 3, 6, 12, and 24 months, all the more so as this category of malignancies presents a risk for local recurrence, follow-up up to 10 years being mandatory.^[5]^ The total disease-specific survival at 5 and 10 years in cases of spermatic cord sarcoma is 75% and 55%.^[23]^

In the present case, following the evolution of the patient after diagnosis and surgical treatment, was not possible because of the occurrence of death within three weeks of the establishment of the diagnosis.

## 4. Conclusion

Although the dedifferentiated liposarcoma of the spermatic cord is a rare entity, whose evolution is unfavorable, extending mainly by contiguity, in this case, the extension of tumor proliferation took place by hematogenous and lymphatic routes, the last ones being rarer ways of metastasis of sarcomas. This aspect is one of the main features of the case. Another defining aspect is the age of onset of the manifestations, which is lower than the average age reported in the literature.

This case highlights the importance of a quick diagnosis, starting with appropriate imaging investigations. The final diagnosis is guaranteed only by corroborating the histopathological and immunohistochemical aspects with the clinical ones. The CISH test has a complementary role, also confirming the diagnosis established in the usual staining. It also draws attention to the unfavorable evolution of sarcomas, which can lead to death through the biochemical effects determined by the occurrence of metastases.

## Author contributions

**Conceptualization:** Mariana Aschie, Gabriela-Izabela Baltatescu.

**Supervision:** Mariana Deacu.

**Validation:** Madalina Bosoteanu.

**Writing – original draft:** Cristian Ionut Orasanu, Raluca Ioana Voda.

**Writing – review & editing:** Sorin Vamesu, Georgeta Camelia Cozaru.
